# Resilient Hospital Design: From Crimean War to COVID-19

**DOI:** 10.1177/19375867231174238

**Published:** 2023-05-10

**Authors:** Kangkang Tang, Bing Chen

**Affiliations:** 1 Department of Civil and Environmental Engineering, Brunel University London, United Kingdom; 2 Department of Urban Planning and Design, Xi’an Jiaotong-Liverpool University (XJTLU), Suzhou, Jiangsu, China

**Keywords:** acute respiratory syndrome coronavirus (SARS-CoV), acute respiratory syndrome coronavirus 2 (SARS-CoV-2), agent-based modeling (ABM), building information modeling (BIM), evidence-based design (EBD)

## Abstract

**Objectives:**

Serious COVID-19 nosocomial infection has demonstrated a need to design our health services in a different manner. Triggered by the current crisis and the interest in rapid deployable hospital, this article discusses how hospital building layouts can be improved to streamline the patient pathways and thus to reduce the risk of hospital-related infections. Another objective of this work is to explore the possibility to develop flexible and scalable hospital building layouts through modular construction. This enables hospitals to better cope with different future demands and thereby enhance the resilience of the healthcare facilities.

**Background:**

During the first wave of COVID-19, approximate one-seventh to one-fifth COVID-19 patients and majority of infected healthcare workers acquired the disease in NHS hospitals. Similar issues emerged during the Crimean War (1853–1856) when more soldiers died from infectious diseases rather than of battlefield casualties in Scutari Hospital. This led to an important collaborative work between Florence Nightingale, who looked into this problem statistically, and Isambard Kingdom Brunel, who designed the rapid deployment Renkioi Hospital which yielded a death rate 90% lower than that in Scutari Hospital. While contemporary medical research and practice have moved beyond Nightingale’s concept of contagion, challenges of optimizing hospital building layouts to support healing and effectively combat nosocomial infections still pose elusive problems that require further investigation.

**Methods:**

Through case study investigations, this article evaluates the risk of nosocomial infections of airborne transmissions under different building layouts, and this provides essential data for infection control in the new-build or refurbished healthcare projects.

**Results:**

Improved hospital layout can be achieved through reconfiguration of rooms and concourse. Design interventions through evidence-based infection risk analysis can reduce congestion and provide extra separation and compartmentalization which will contribute the reduced nosocomial infection rate.

**Conclusions:**

A resilient hospital shall be able to cope with unexpected circumstances and be flexible to change when new challenges arise, without compromising the safety and well-being of frontline medical staff and other patients. Such an organizational resilience depends on not only flexible clinical protocols but also flexible hospital building layouts. The latter allows hospitals to get better prepared for rapidly changing patient expectations, medical advances, and extreme weather events. The reconfigurability of an existing healthcare facility can be further enhanced through modular construction, standardization of building components, and additional space considered.

## Overview of Hospital Infection Control Design

**Figure 1.**
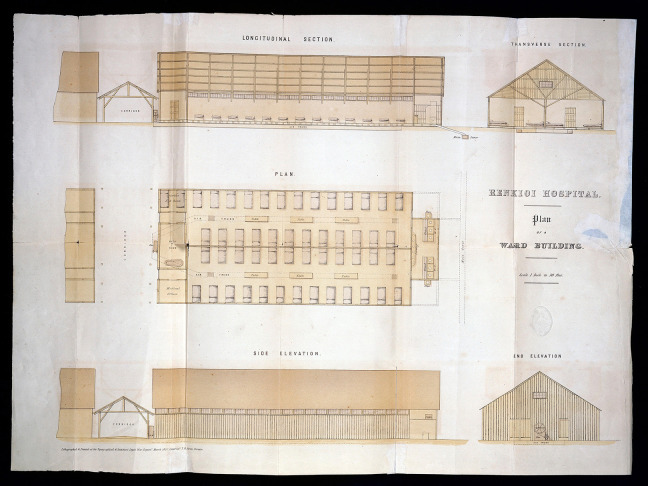
Renkioi Hospital—Standard ward layout. *Source*: Courtesy of Wellcome Collection (Alexander, 1857).

**Figure 2.**
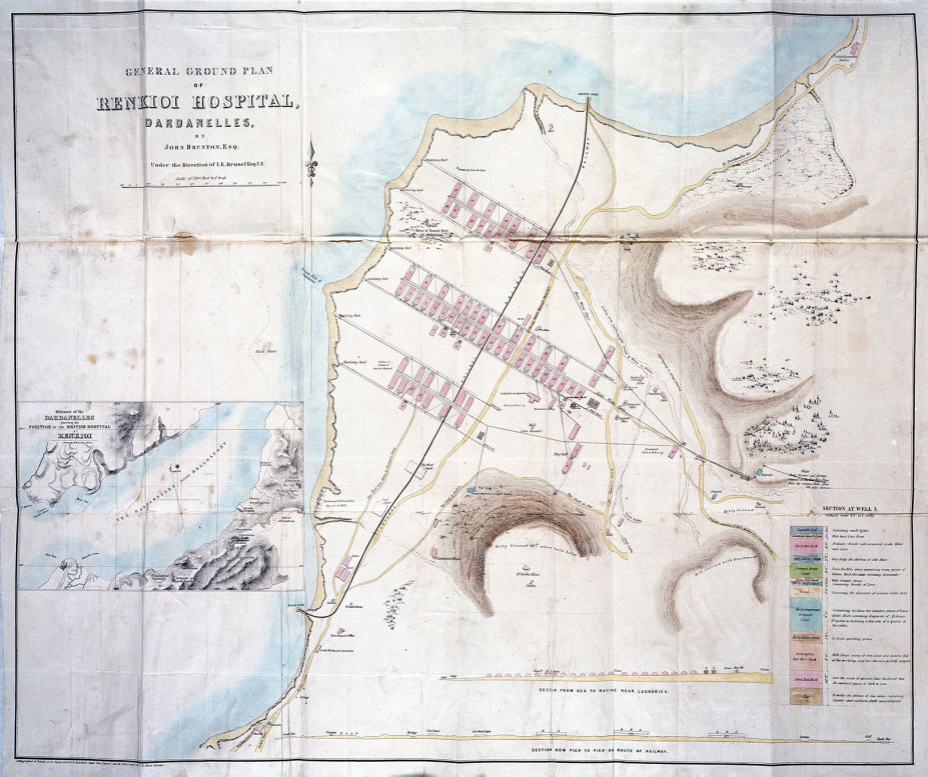
Renkioi Hospital—Site plan. *Source*: Courtesy of Wellcome Collection (Alexander, 1857).

**Figure 3.**
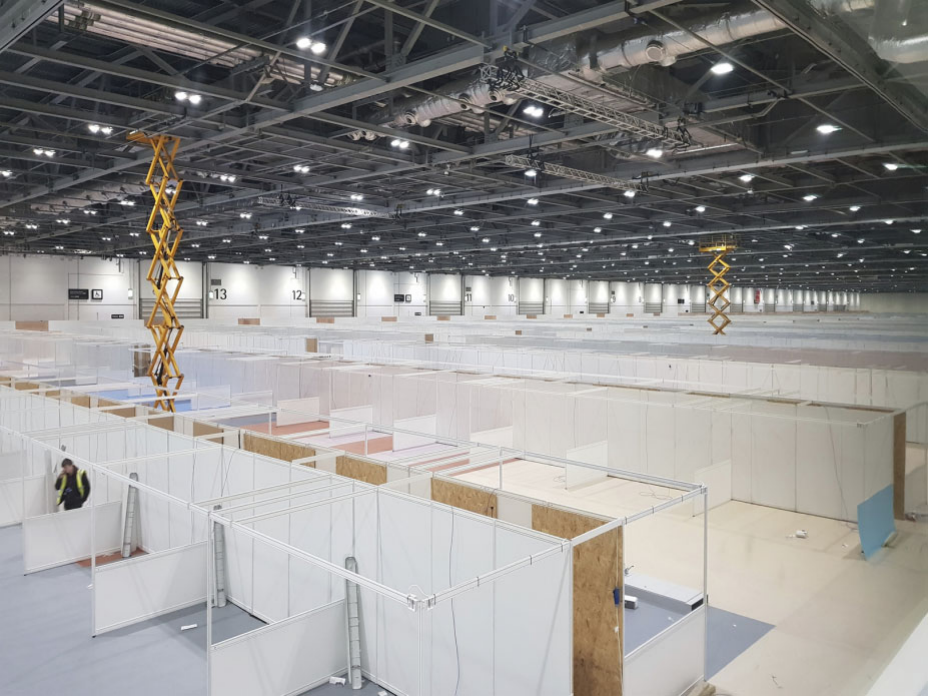
London ExCel Nightingale Hospital—Construction stage. *Source*: Images used with kind permission from BDP.

**Figure 4.**
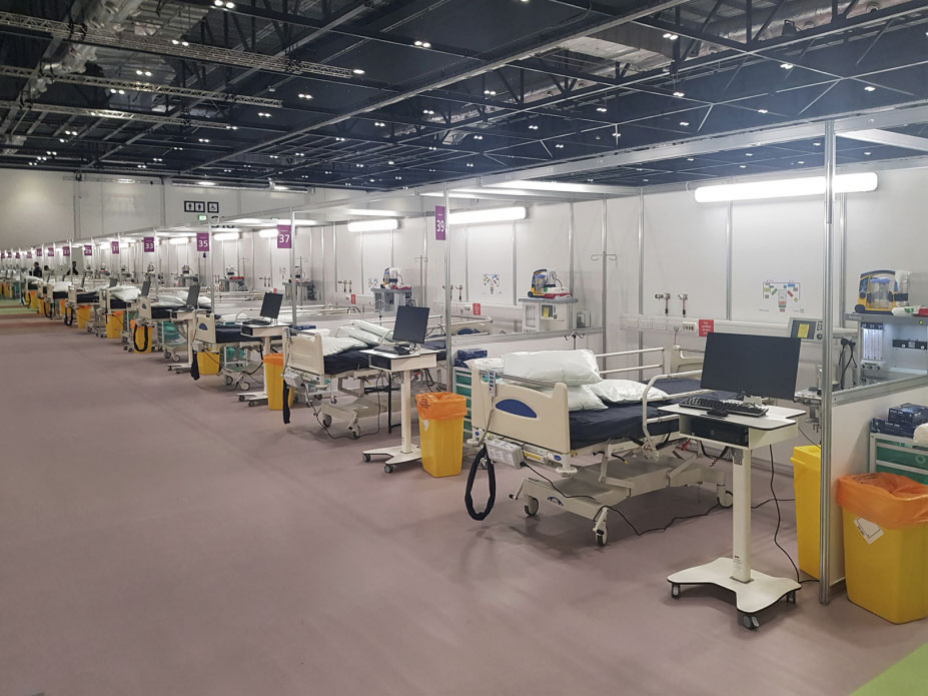
London ExCel Nightingale Hospital—Handover and close out. *Source*: Images used with kind permission from BDP.

**Figure 5.**
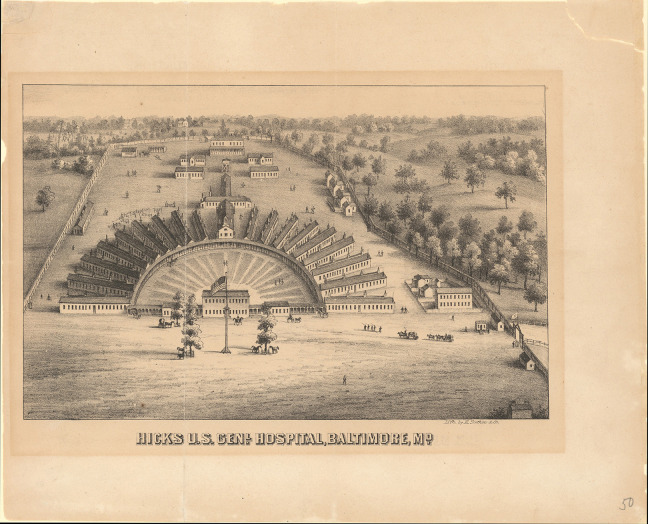
Hicks U.S. General Hospital, Baltimore, MD (1864). *Source*: Courtesy of Enoch Pratt Free Library, Maryland’s State Library Resource Centre.

In the winter of 1854, over 4,000 soldiers died in Scutari Hospital during the Crimean War (1853–1856) and majority of them died from infectious diseases rather than of battlefield casualties. This led to an important report by Florence Nightingale, who looked into the iatrogenic problem statistically, and the Renkioi Hospital designed by Isambard Kingdom Brunel in 1855 (Silver, 2004). Renkioi Hospital was based on a standardized two-ward hospital building and each ward has 25 patient beds (Figure 1). One of the major design features of Renkioi Hospital is its ability to “expand.” Owing to Brunel’s resilient design concept based on prefabrication and modular construction, the total number of patient beds increased from 300 to 1,500 within a few months as seen in Figure 2 (Silver, 2004). The similar design principle and building techniques can be seen in the construction of the London ExCel Nightingale Hospital in 2020 (Figures 3 and 4), and its first phase of the construction process took only 9 days to achieve an initial capacity of 500 patient beds (BBC, 2020; NHS, 2020). Another important legacy of Renkioi Hospital is the pavilion style of building layouts: Three rows of buildings were set out onsite with ample spaces between them to promote natural ventilation. Such a pavilion-style hospital successfully prevented the spread of infectious diseases and yielded a death rate 90% less than that in Scutari Hospital (FNM, 2020). Renkioi Hospital also set new standards for hygiene, access, and space. By the 1860s, low-rise pavilion-style hospitals had become the most favorable hospital option in Europe and America. Similar design principles had been followed in the barracks hospitals during the American Civil War (1861–1865; Kisacky, 2017). An example of this trend can be seen in the Baltimore Barracks Hospital, which was a one-story facility laid out in a circular array (Figure 5). Its innovative design not only facilitated natural ventilation but also enabled a more efficient logistics system due to its centripetal layout.

**Figure 6.**
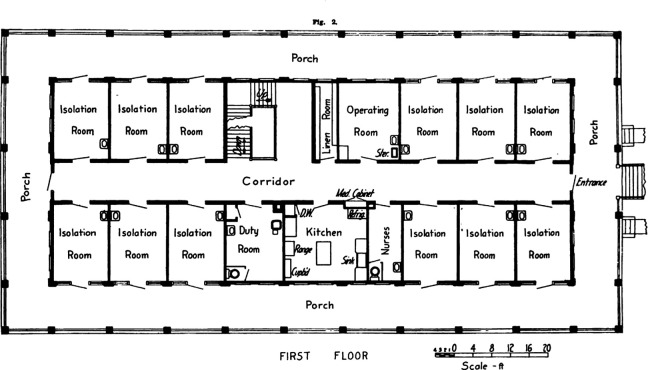
Contagious Hospital, University of Michigan (1914). *Source*: Courtesy of Kara Gavin, Michigan Medicine Department of Communication (University of Michigan, 1914).

The significant advancements in the understanding of germs (microorganisms) by the late 1880s had introduced revolutionary changes in surgical practices. As a result, many operating theaters had been designed with meticulous attention to aseptic standards (Crellin, 1966). In 1914, the University of Michigan established its Contagious Hospital, implementing more stringent aseptic techniques and comprehensive isolation strategies for improved management of contagious diseases. Aseptic barriers include isolation rooms which were 12 feet wide and 16 feet long with two doors: One was opened into the corridor, and another led to the terrace (Figure 6). This enabled a one-way traffic system within the hospital, enabling patients to exit without having to retrace their steps through the same corridor. Additionally, visitors could communicate with patients from the terrace without needing to enter the isolation rooms, ensuring infection control measures were maintained. In the early 20th century, one-way traffic system has been popularly used in hospitals to optimize workforce management and prevent the spread of infectious diseases. In the United States, City Isolation Hospital in Minneapolis was designed in such a way that “a person enters the soiled room and the door cannot be opened to let him pass back into the ward. He has to go through the whole (decontamination) process and out the other way” (Kisacky, 2017). Mechanical extract ventilation system has also been developed in a revolutionary way and negative-pressure ventilation system has been used in many hospitals to prevent infected air from an infectious disease area entering into a protected area. The success of pavilion-style hospitals continued until the turn of the 20th century. Another important feature of Renkioi Hospital, that is, its expandability, has inspired the design of other hospitals, allowing them to cope with a sudden surge of patient numbers under various situations (Kisacky, 2017).

The discovery of penicillin in 1928 marked the beginning of the golden age of antibiotics, which reached its peak in the mid-1950s. Antibiotics, in conjunction with more stringent surgical aseptic procedures, overshadowed the principles of disease prevention through natural ventilation and sunlight as advocated by Nightingale. This led to the development of vertical and factory assembly-line-style hospitals that prioritized cutting healthcare and management costs. For example, the Bellevue Hospital in New York was designed as a 25-storey high-rise building, with interior wards lacking direct access to fresh air and sunlight (Carmody, 1973). Misuse and overuse of antibiotics however led to drug-resistant pathogens and severe antimicrobial resistance (AMR). In the 1970s, the problem of AMR infections was so serious that many hospitals had to be temporarily closed (Kisacky, 2017). Similar challenges were faced by many Accident and Emergency (A&E) departments in the UK during the outbreak of the COVID-19 pandemic in 2020 (Lee et al., 2021). Substantial studies have shown the relationship between ventilation and airborne transmission of infectious diseases like influenza and tuberculosis (Yau et al., 2011). The current UK Health Technical Memorandum HTM 03-01 (DHSC, 2021) also states that “the default method of ventilation should as far as possible be natural ventilation” which is well in line with Nightingale’s ideal hospitals as discussed in her “Notes on Hospitals” (Nightingale, 1863). Drawing on the lessons from historical challenges with AMR infections and the more recent crisis of nosocomial COVID-19 infections, it is evident that hospital design plays a crucial role in preventing disease transmission and safeguarding public health.

## Impact of COVID-19 on Hospital Design

Modern hospital design philosophy is predominantly based on the evidence-based design (EBD) approach which is a scientific methodology that emphasizes the conscientious, explicit and judicious use of data acquired in the past to critically inform the present design decisions and, in the meantime, collect further data through postoccupancy evaluation process to further enrich the evidence database (Phiri & Chen, 2014). During the SARS-CoV outbreak in 2003, over 20% of the confirmed cases were attributed to nosocomial transmissions, including most of the frontline healthcare staff (Ho et al., 2003; Xiao et al., 2020). The NHS gained valuable insights from this experience, underscoring the significance of isolation units in effectively managing highly contagious patients and containing the spread of infectious diseases (Van Enk, 2006). Single-bed rooms with en-suite sanitary facilities have been identified as a crucial measure in reducing the risk of nosocomial infections (Brouqui et al., 2009). Despite these previous efforts, serious nosocomial transmissions still took place amid the outbreak of the COVID-19 pandemic in 2020. Approximate one-seventh to one-fifth COVID-19 patients and majority of infected healthcare workers acquired the disease in NHS hospitals during the first wave (Campbell and Caelainn, 2021; Evans et al., 2020; Read et al., 2021). Rickman et al. (2020) investigated nosocomial COVID-19 transmission cases in London hospitals and reported that 55% of the patient-to-patient infections took place in the same bay; 14% were in different bays but on the same floor; and 12% of infections were found to have occurred in single-occupancy rooms despite their presumed lower risk of transmission. In comparison to NHS hospitals, nosocomial transmissions of COVID-19 in care homes and mental health hospitals were even worse: The nosocomial infection proportion in the UK residential community care hospitals were estimated to be 61.9%, and 67.5% in mental health hospitals (Read et al., 2021). Healthcare Safety Investigation Branch (2020) attributed serious COVID-19 nosocomial transmissions to the design of hospitals, and highlighted the importance to “reconsider the design of ward work systems and equipment layout to mitigate the risk of nosocomial transmission”.

***reconsider the design of ward work systems and equipment layout to mitigate the risk of nosocomial transmission***

Another lesson from the COVID-19 pandemic concerning hospital resilience was the unexpected shock created by an unprecedented number of patients who required admission to hospitals and especially the intensive care units (ICUs). Many critically ill patients had to be treated outside the ICUs, while NHS hospitals were struggling to maintain the standards of care under the strain. The strain on NHS hospitals was compounded by poorly designed hospital facilities, which impacted patient triage and flow processes, exacerbating the issue of long patient waiting hours, amid staff shortages and funding deficiencies. In January 2020, about 15% of all patients visiting A&E in England had to wait for over 4 hr to be admitted, transferred, or discharged. This figure had risen significantly to over 28% by January 2023 (NHS, 2023). COVID-19 has brought well-known problems to the forefront at scale, and it is perhaps more important than ever to rethink Renkioi Hospital’s legacies when designing and setting out health and care services. By finding solutions to these challenges, we will not only be resolving immediate concerns but also paving the way for enduring solutions that will benefit generations to come.

## Infection Risk Assessment and Epidemiological Modeling

The design of Renkioi Hospital was based on the serious interaction between healthcare and engineering. Modern hospital infection risk evaluation is an area where engineering and healthcare research overlaps around the built environment. One example of this convergence is the utilization of computational fluid dynamics (CFD) modeling, which allows for the simulation of air flow patterns and the identification of the potential hotspot areas of contamination within a healthcare facility (Mohamadi & Fazeli, 2022). It provides critical evidence for the development of infection control protocols and facility design improvements, effectively mitigating the risk of infection transmission. Additionally, the utilization of epidemiological analysis based on CFD modeling provides valuable insights into the transmission dynamics of infectious diseases and enables extrapolation of infection rates over time, aiding in a comprehensive understanding of the disease’s spread.

The Wells–Riley equation developed by William Wells and Richard Riley (Aganovic et al., 2021) has been extensively used to assess the risk of indoor transmission of airborne diseases such as influenza, TB, and measles. Since the outbreak of COVID-19, it has been used to model the transmission of the SARS-CoV-2. The Wells–Riley equation can be written as:


1 $$P_{I}\;=\;1-\text{exp}\left(-\frac{Iqpt}{Q}\right),$$

where *P_I_* is the probability of infection (%), *I* is the number of infectors, *p* is the pulmonary ventilation rate of the susceptible (m^3^/h), *q* is the quanta generation rate of patients (quanta/h), *t* is the exposure time (h), and *Q* is the room ventilation rate (m^3^/h). It should be noted that *q* is a hypothetical unit which cannot be directly obtained through experiments. The outbreak of influenza on a commercial aircraft in 1999 led to 20 passenger infections (of 74 passengers in total), indicating a 27% infection rate (*P_I_*; Marsden, 2003). Considering the standard aircraft cabin air exchange rate and flight hours (*t*), *q* was quantitatively decided, and this enables the evaluation of the risk of outbreak of a similar airborne disease under different environmental conditions (Marsden, 2003).

Tang and Chen (2021) determined the *R_0_* (i.e., the average number of secondary cases that results from the index patient) of COVID-19 infections in a hospital using agent-based modeling (ABM). ABM is a form of computational model for simulating the actions and interactions of individuals (agents). In the context of healthcare research, ABM has been used model the spread of infectious diseases and evaluate the effectiveness of diverse nonpharmaceutical interventions (Hoertel et al., 2020; Mahmood et al., 2020; Maziarz and Zach, 2020; Tang & Chen, 2021). There is however still lack of evidence to assert the adequacy of ABM and especially lack of sensitivity (parametric) studies based on the real hospital data (including environmental conditions) to verify the computer simulation results. As a result, evidence obtained through ABM has not been considered in the assessment of healthcare interventions during the pandemic by the Oxford Centre for Levels of Evidence (OCEBM, 2009) or the National Institute for Health and Care Excellence Guidelines (NICE, 2014).

## Research Gaps

Medical research and practice have moved beyond Nightingale’s concept of contagion, and the design of hospitals has responded to the new theories and practice with time to prevent nosocomial infections. This includes the implementation of measures such as antiseptics, asepsis, antibiotics, management of antibiotic-resistant strains, and the incorporation of epidemiological approaches. Over the years, there has been a significant amount of research dedicated to understanding the effects of healthcare settings on morbidity and mortality associated with nosocomial and iatrogenic infections. Despite these notable advancements, the progress has been limited in comparison to the pioneering work of Florence Nightingale in harnessing engineering principles to tackle similar issues. There is still a lack of studies to justify the efficacy of design interventions through evidence-based infection risk analysis. Other research gaps concerning the organizational resilience of a hospital became obvious amid the pandemic:

What would a resilient hospital look like during an outbreak?
What is the role of hospital design, for example, building layouts and single room provision, in relation to infection prevention and control?

Hospitals are normally designed for average patient loads, not for a sudden surge of patients as witnessed during the pandemic. What design tools can be used to inform better and more resilient health and care services? Can modern methods of construction help to transform health and care beyond current hospital settings and still manage unintended consequences on built environment risks?

## Research Methodology

In this study, infection risk assessment based on epidemiological modeling was conducted through case study investigations. The nosocomial infection risk of a clinical infection unit (CIU) was assessed using ABM, taking into account the intricate spatially explicit stochastic simulations within a virtual hospital built environment. This was achieved by superimposing an epidemiological model for transmission (or the Wells–Riley model) on a hospital building model with a constant infection risk factor (*P_I_*) assumed. The hospital building model was created using building information modeling (BIM) software Revit (Autodesk, 2020). The Revit model was saved as an Industry Foundation Classes (IFC) file which is a standard format for exchanging BIM information across different software. The IFC file was then imported into agent-based simulation software MassMotion (OASYS, 2020) with geometrical information of all structural and nonstructural elements. The pathway of each patient was determined based on a minimum agent time cost model (Equation 2; Kinsey, 2015), which predicted their trajectory through the hospital setting.


2 $$Agent\;time\;cost\;=W_{D}\;\times \;\left(\frac{D_{G}}{V}\right)\;+W_{q}\;\times Q+W_{L}\times L,$$

where:

*Cost*: Perceived total travel time alone the route (s),

$W_{D}$ : Distance weight of each person,

$D_{G}$ : Total distance from the person’s present position to the destiny (m),

V : Velocity of each person (m/s),

$W_{q}$ : Queue weight of each person,

Q : Expected time in queue before reaching a particular point (s),

$W_{L}$ : Geometric component traversal weight, and

L : Geometric component type cost (s).

## Case Study Investigation

**Figure 7.**
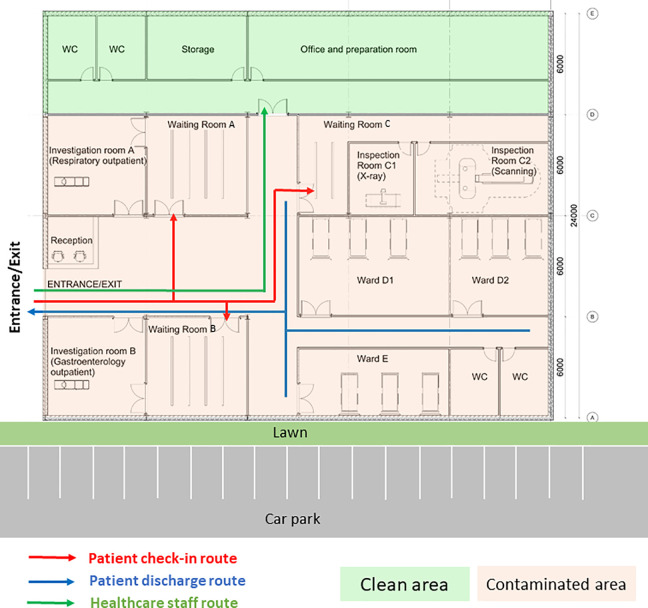
Clinical infection unit, Layout A.

In order to justify the efficacy of design interventions on the risk of nosocomial infections, a case study investigation of a CIU was conducted. The CIU is managed by the infectious disease team and its building layout is shown as Figure 7. CIU provides specialist services including outpatient clinical diagnosis and inpatient care. It consists of different functional units including consultation rooms, surgeries, ward units, MRI, and diagnostic rooms. Patients are predominantly admitted through internal referrals from A&E and Urgent Treatment Centers. The evaluation of the CIU will build confidence to model large-scale general hospital clinical processes where ongoing research is being conducted.

**Figure 8.**
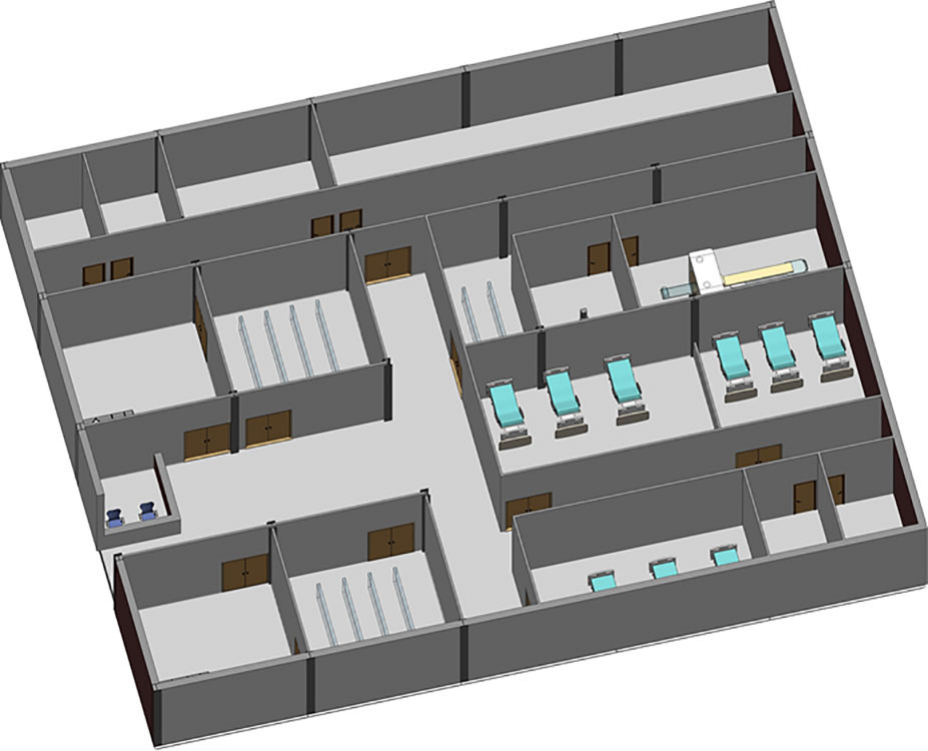
Clinical infection unit, Layout A isometric view.

**Figure 9.**
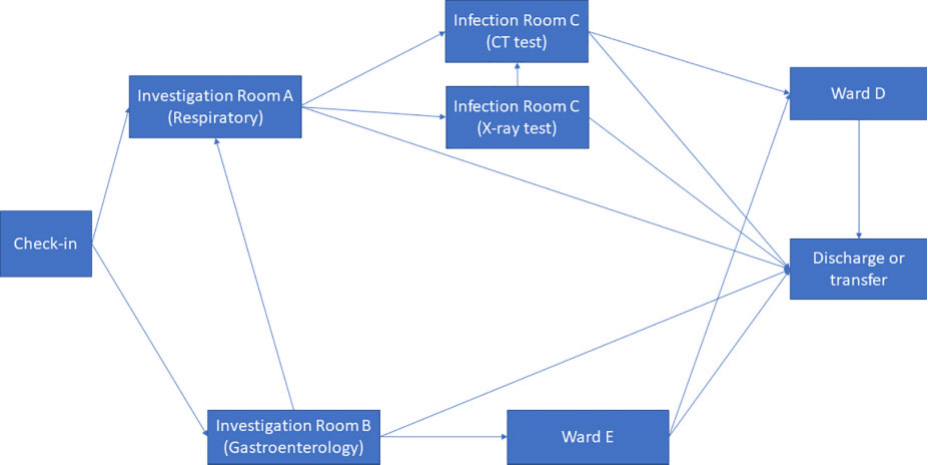
Clinical process.

The CIU building has an orthogonal column gridlines 6 m × 6 m. The virtual model of the facility was created using Autodesk Revit (Autodesk, 2020). The Revit model was then saved as the IFC file that was imported into ABM software MassMotion (OASYS, 2020). All structural and nonstructural elements including furniture, partition walls, and heavy medical instruments were modeled as “obstacles” in the ABM model (Figure 8). Two hundred patients (agents) were admitted in the CIU within 30 min. Forty percent of the patients were to seek medical help on digestive problems, and 60% had developed respiratory distress syndromes. No accompanying visitors for patients were considered in the computer simulation. Medical staffs and supporting staffs were not considered in ABM either. The clinical process is schematically shown in Figure 9. All patients walked at an average speed of 1.35 m/s, with 95% of the distribution within 0.25 m/s standard deviations of the average speed. The velocity at which patients move is also influenced by the density of individuals in close proximity, as it impacts their ability to navigate through the environment. Additionally, each patient dynamically selects their path inside the hospital based on the real-time minimum time cost (or Equation 2), taking into account factors such as congestion and distance to optimize their movement in the moment.

In the epidemiological analysis, five COVID-19 patients (agents) were admitted at following time. The simulation of airborne transmission of (SARS-CoV-2) pathogens was conducted using a superimposed infection model. In this work, airborne transmissions between patients in the waiting rooms were considered based on an assumption of 10% probability of infection (*P_I_*).

Patient 1 visits Investigation Room A at 5 min (with respiratory disease symptoms).
Patient 2 visits Investigation Room A at 10 min (with respiratory disease symptoms).
Patient 3 visits Investigation Room A at 15 min (with respiratory disease symptoms).
Patient 4 visits Investigation Room B at 5 min (with digestive disease symptoms).
Patient 5 visits Investigation Room B at 10 min (with digestive disease symptoms).

**Figure 10.**
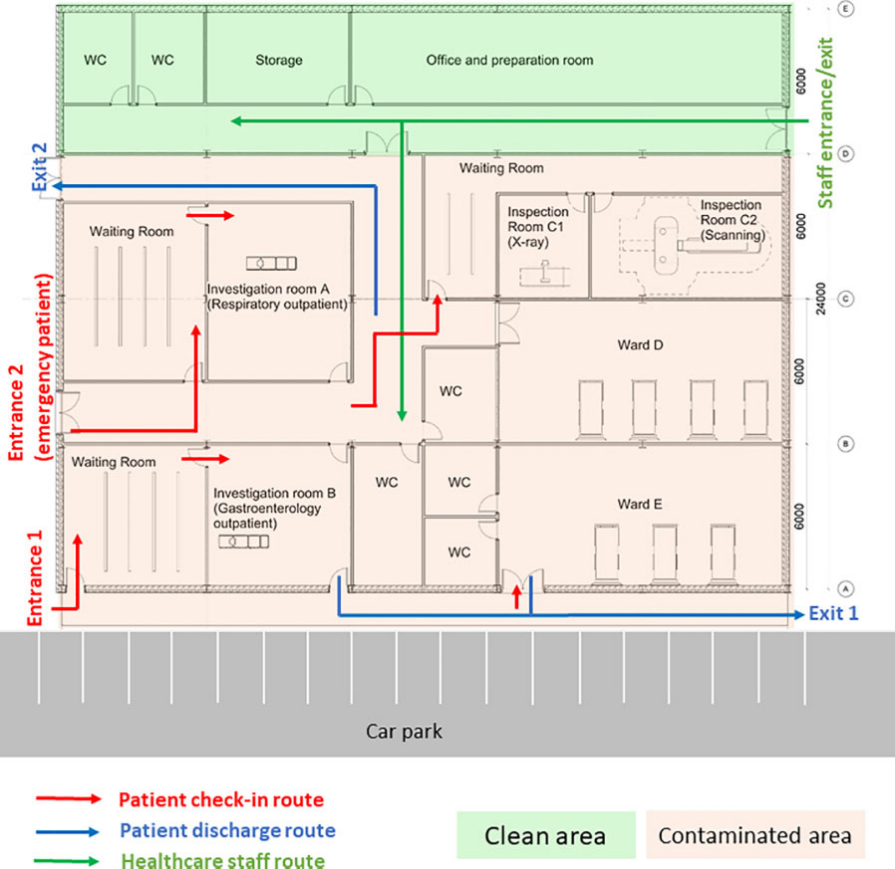
Improved clinical infection unit, Layout B.

During the first wave of COVID-19, the Care Quality Commission (CQC) reported that at least one half of UK hospitals have not got suitable designated pathways to allow patients with symptomatic COVID-19 to be safely admitted to and discharged from hospitals (CQC, 2020). Owing to the divisible sections in the original design (Layout A), an improved CIU Layout B was developed based on the same column gridlines (e.g., 6 m × 6 m) but with reconfiguration of rooms and concourse as seen in Figure 10. Design B facilitates a one-way patient traffic system by incorporating two separate entrances, additional separation, and compartmentalization within the healthcare facility. Separate entrances were achieved by establishing a new patient pathway on the greenfield of the original Design A (Figure 8). The conversion of investigation rooms, waiting rooms, and wards has enabled the creation of dedicated rooms specifically designed for suspected or confirmed COVID-19 patients. This modification has been implemented to ensure appropriate isolation and care for individuals with potential or confirmed cases of the virus within the healthcare facility. Same as the previous epidemiological analysis, 200 agents (patients) were admitted in the facility within 30 min, including five COVID-19 patients for the epidemiological analysis.

## Results and Discussion

**Figure 11.**
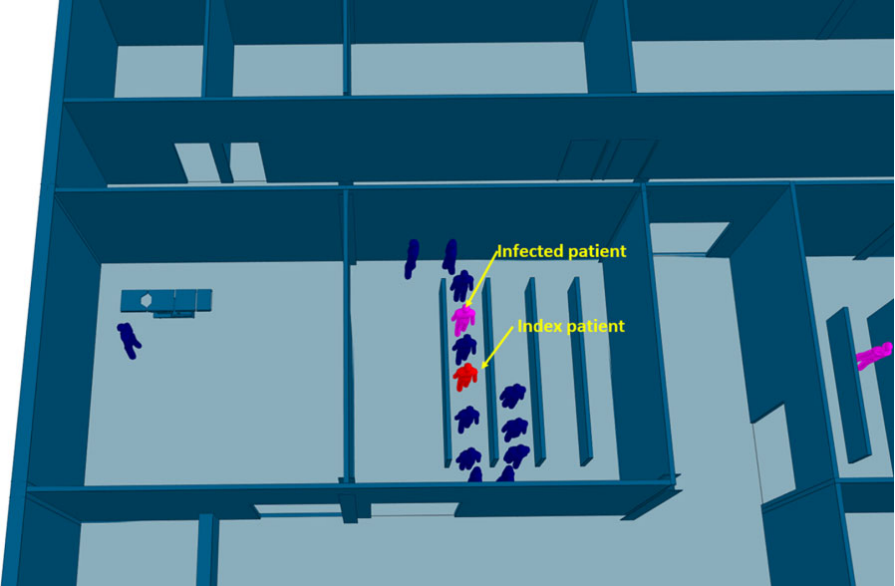
Agent-based modeling results (clinical infection unit, Layout A)—Nosocomial infection in Investigation Room A.

**Figure 12. fig12-19375867231174238:**
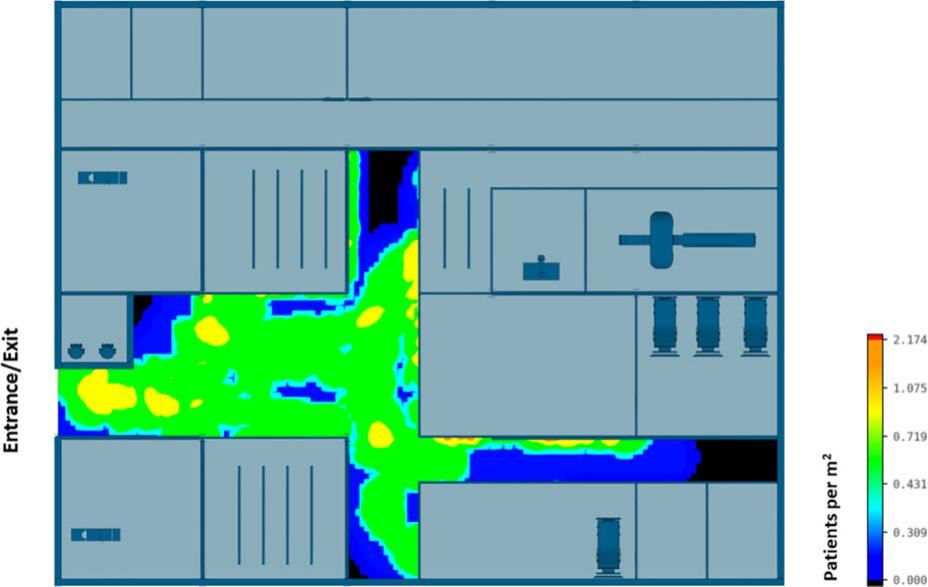
Agent-based modeling results (clinical infection unit, Layout A)—Concourse congestion analysis.

**Figure 13.**
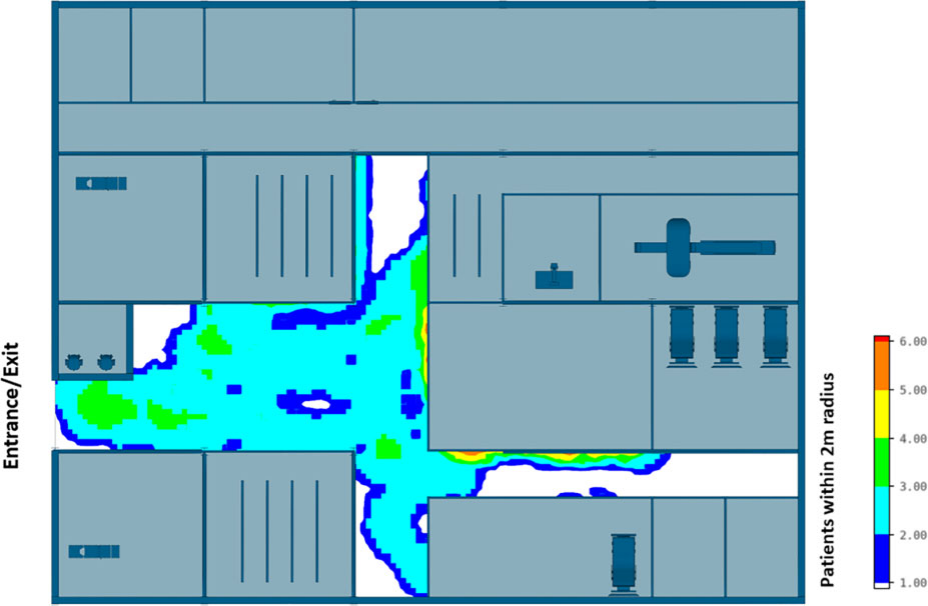
Agent-based modeling results (clinical infection unit, Layout A)—Maximum number of patients within 2 m radius.

The efficiency of different hospital building layouts (A and B) was assessed based on the epidemiological analysis, and the results are schematically shown in Figures 11 [Fig fig12-19375867231174238]–13. Figure 11 illustrates the potential for nosocomial infections in close proximity to COVID-19 patients. The figure provides visual representation of the potential risk of infection transmission in the immediate vicinity of patients with COVID-19 in a healthcare facility. The findings from the ABM indicate that of 195 suspected patients, 16 patients may contract the disease when five COVID-19 patients are admitted to the facility, resulting in an infection rate of 8.2%. These ABM results provide insights into the potential spread of the disease among suspected patients in the healthcare facility. The infected cases include 13 patients in the Waiting Room A and three patients in the Waiting Room B. Considering SARS-CoV-2 can be transmitted through droplets and aerosols, the distance between patients plays a critical role in influencing risk perception. The proximity or distance between individuals is a significant factor that can impact the perception of the risk of transmission of the virus. Figures 12 and 13 show severe pedestrian congestion in the concourse of the hospital building, potentially resulting in a breach of social distancing rules (2 m distancing) and increasing the risk of nosocomial transmission of the disease. The image illustrates the potential challenges in maintaining adequate social distancing measures in the hospital concourse, which may contribute to the spread of the virus within the healthcare facility.

**Figure 14.**
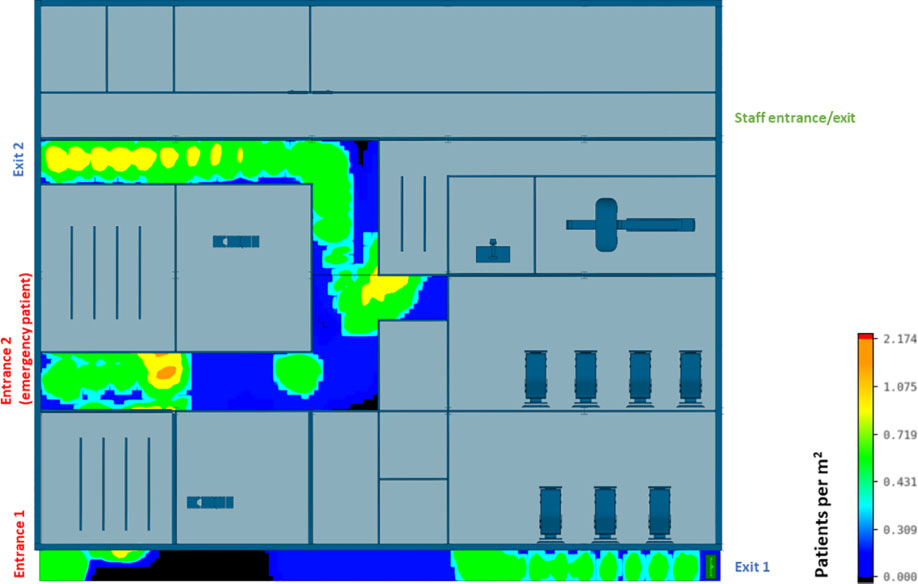
Agent-based modeling results (clinical infection unit, Layout B)—Concourse congestion analysis.

**Figure 15.**
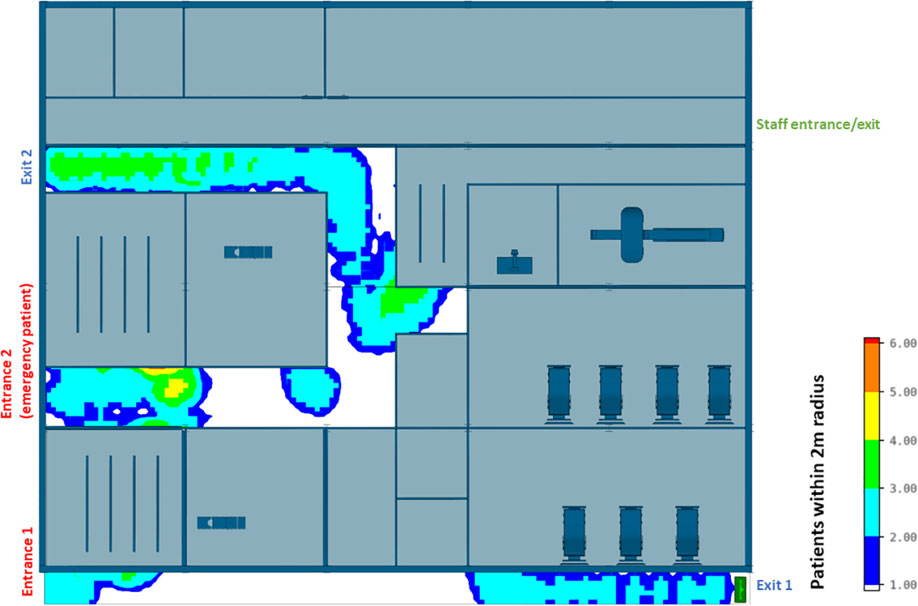
Agent-based modeling results (clinical infection unit, Layout B)—Maximum number of patients within 2 m radius.

A well-insulated reception has been introduced in CIU design B. Figures 14 and 15 show that the updated hospital building layouts effectively reduced congestion in the corridor in comparison to Figures 12 and 13 (e.g., reduced number of patients per square meter). The introduction of patient flow separation measures, along with streamlined workflows, has resulted in a notable reduction in the infection rate compared to the original design. With the admission of five COVID-19 patients, only 12 of 195 patients met the criteria for nosocomial infections, showcasing a decreased infection rate of 6.1%. These include nine infected patients in the Waiting Room A, one patient in the Waiting Room B, and two patients in the Waiting Room C.

**Figure 16.**
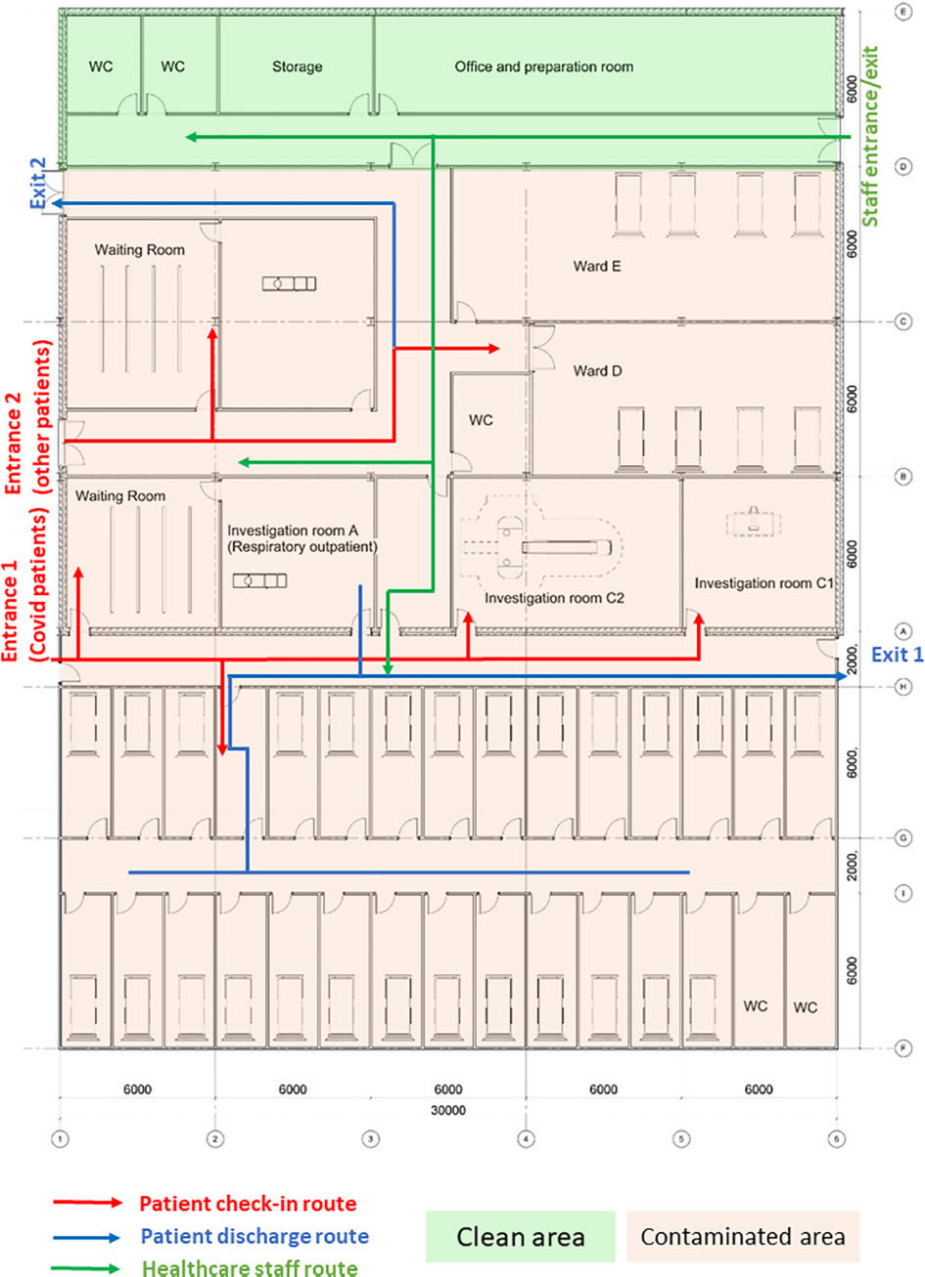
Improved clinical infection unit (Layout C) with “pop-up” triage wards.

Figure 16 shows the surge capacity of the CIU can be further enhanced with integrated “pop-up” triage wards which can be “plugged into” the existing hospital on its car park. Supporting units including waste proposal, plant rooms, and service support can be integrated into the pop-up triage system. It should be noted that such reconfiguration ability is only possible if a hospital is “retrofit ready,” or different programmatic layouts like Figure 16 have been considered at the design stage. In this case study, the hospital’s expandability is ensured through the inclusion of additional space, such as the greenfield and car park, in the original design. The adaptation of the pop-up facilities can be further facilitated if removable external walls and lightweight internal partitions are used in the original design. For a more permanent renovation or refurbishment, modular construction can be used to facilitate the construction procedure. Modular construction is based on mass production of factory-built modules to speed up the construction process. Modular units, such as prefabricated walls and floors, can be used in the construction of buildings. These units are manufactured offsite and then assembled onsite, allowing for a fully functional hospital building to be transported to the site and connected to an existing building. For example, George Eliot Hospital acquired a new 30-bed ward which comprises over 30 modular units with integrated medical facilities such as medical gas piping systems, access controls, fire escape ramps, and nurses’ stations during the pandemic. The use of prefabricated modular units allowed for a significant reduction in the construction schedule, from 20 weeks to 14 weeks, resulting in a more efficient and timely response to the increased demand for medical facilities.

## Conclusion

During the COVID-19 pandemic, serious nosocomial infections have been attributed to contaminated hospital indoor environments, which necessitates further multidisciplinary studies to better understand the dynamics of disease transmission in healthcare settings. Florence Nightingale’s belief in the critical role of hospital design in preventing infections remains relevant in the context of the current crisis, highlighting the importance of creating healthcare facilities that are designed with infection prevention in mind. Furthermore, the example of Brunel’s design for Renkioi Hospital, which allowed for offsite prefabrication and onsite assembly, showcases the potential of innovative design approaches to meet urgent healthcare needs efficiently. These examples also illustrate the ongoing significance of hospital design in the management of infectious disease outbreaks and the potential for innovative solutions to enhance healthcare facilities’ resilience.

Modern methods of construction have moved beyond Brunel’s concept of rapid deployable design, but problems concerning future flexibilities or the ability to be adaptable to future changes still remain. This article reviews the hospital-based intervention strategies and discusses the possibility to develop more resilient hospital building layouts to better control nosocomial infections through computer simulations. The overarching goal of computer simulation, as discussed in this work, was not to make real life more predictable but rather to demonstrate that the organizational resilience of a healthcare facility can be enhanced through reconfigurability, enabling it to adapt effectively to changing future demands. The present world is highly interconnected, and deadly pathogens can spark global pandemic in a few weeks or days. This, in conjunction with the changing demographic of patients, will have a transformative impact on design interventions and healthcare resilience. A resilient hospital shall be able to cope with such unexpected circumstances and be flexible to change when new challenges arise, without compromising the safety and well-being of frontline medical staff and other patients. Such an organizational resilience depends on not only flexible clinical protocols but also flexible hospital building layouts. The latter allows hospitals to get better prepared for rapidly changing patient expectations, medical advances, and extreme weather events. The reconfigurability of an existing healthcare facility can be further enhanced through modular construction, standardization of building components, and additional space considered at the design stage. It is hoped that some findings from this research, after being further verified via real-life experiments, would be used as evidence to inform future hospital designs, the development of tools in healthcare, and future technical guidance on infection control and prevention.

## Implications for Practice

- Modern methods of construction have moved beyond Brunel’s concept of rapid deployable design, but problems concerning future flexibilities or the ability to be adaptable to future changes still remain.
- Serious nosocomial infections during the outbreak of the COVID-19 pandemic are implicated in the contaminated hospital indoor environments which requires further multidisciplinary studies.
- The reconfigurability of an existing healthcare facility can be further enhanced through standardization of building components to maximize compatibility and to facilitate the exchange of knowledge and information on asset management.
- It is expected some findings from this research would be used as evidence to inform future hospital designs and the further development of the simulation tools, and the technical guidance and tools in health and care.
